# Numerical Study on the Cavitation Characteristics of Micro Automotive Electronic Pumps under Thermodynamic Effect

**DOI:** 10.3390/mi13071063

**Published:** 2022-07-01

**Authors:** Kaipeng Wu, Asad Ali, Changhong Feng, Qiaorui Si, Qian Chen, Chunhao Shen

**Affiliations:** 1 Research Center of Fluid Machinery Engineering and Technology, Jiangsu University, Zhenjiang 212013, China; siqiaorui@ujs.edu.cn (Q.S.); 2222011062@stmail.ujs.edu.cn (K.W.); 5103190316@stmail.ujs.edu.cn (A.A.); shenchunhaoujs@163.com (C.S.); 2 Feilong Auto Components Co., Ltd., Nanyang 474500, China; 3 School of Electrical and Information Engineering, Jiangsu University, Zhenjiang 212013, China; chenqian0501@ujs.edu.cn

**Keywords:** micro automotive electronic pump, computational fluid dynamics, numerical simulation, experimental techniques, cavitation, thermodynamic effects

## Abstract

In order to study the influence of thermodynamic effects on the cavitation performance of hydromechanics, the Singhal cavitation model was modified considering the influence of the thermo-dynamic effects, and the modified cavitation model was written into CFX using the CEL language. Numerical simulation of the cavitation full flow field at different temperatures (25 °C, 50 °C and 70 °C) was carried out with the automotive electronic water pump as the research object. The results show that the variation trend of the external characteristic simulation and experimental values is the same at all flow rates, and the calculation accuracy meets the subsequent cavitation demand. With the increase in temperature, the low-pressure area inside the automotive electronic pump’s impeller decreases. NPSHr decreases and the cavitation resistance is enhanced. During the process of no cavitation to cavitation, the maximum pressure pulsation amplitude in the impeller channel gradually increases. The generation and collapse of cavitations cause the change of pressure pulsation in the internal flow field, causing pump vibration.

## 1. Introduction

Automotive electronic pumps are core components of internal combustion engine cooling systems for drainage and irrigation products, which need to be operated at a high temperature and high speed for a long time. Because the physical parameters of coolant change with temperature, it is easy to induce cavitation of the pump and then affect the stable operation of the system. Cavitation is a flow phenomenon involving a phase change process in which the thermodynamic effect exists during the occurrence, development, and collapse of the bubble [1,2,3,4,5]. As the vaporization medium needs to absorb heat from the surrounding liquid, the temperature will have a certain effect on the physical parameters of the medium, which in turn affects the process of heat exchange during the vaporization process. Therefore, the existing cavitation model should be appropriately modified to predict the cavitation phenomenon accurately, and the source term considering the thermodynamic effect should be added.

Many scholars have studied cavitation flow under thermodynamic effects. In terms of experiments, Fruman et al. [6] proposed a cavitation correction model considering cavity heat transfer by analyzing the heat transfer process in the cavitation process, using R114 as the test object, and found that the cavitation performance improved with the increase of temperature. Franc et al. [7] made a cavitation test of Freon R-114 flowing through the induced wheel at different temperatures and investigated the effect of temperature on the thermodynamic effect of significant fluid cavitation. By comparing the experimental phenomena of different temperature water around NACA0015 hydrofoil at the same cavitation number, Cervone [8] found that the difference in the water vacuole size was not significant at 293 K and 323 K; however, the vacuole length decreased significantly at 343 K and gave a reasonable explanation for this phenomenon. Yoshida et al. [9,10] systematically investigated the thermal effect on the cavitation performance and cavitation instability of the induced wheel by using liquid nitrogen as the working medium. Gustavsson et al. [11,12] carried out a cavitation flow test of water and fluorinated ketone around NACA0015 hydrofoil separately and found that the cavitation length of fluorinated ketone was shorter than that of water at the same cavitation number and the cavitation thermal inhibition effect was more obvious.

With the development of computer technology and computational fluid dynamics [13,14,15], numerical simulation has become an essential tool for studying cavitation problems. Deshpande et al. [16] built on the Navier-Stokes equation with compressibility and pseudo-time-step progression by coupling the energy equation, and developed a thermodynamic model applicable to the cavitation of low-temperature fluids. Franc et al. [17] performed an analysis founded on the Rayleigh-Plesset equation, modified the cavitation model with temperature as one of the influencing factors of cavitation, and validated it accordingly. Hosangadi et al. [18] described a compressible multiphase flow formulation to account for the energy balance and variable thermodynamic properties of the fluid, identified fundamental changes in the physical characteristics of the cavity when thermal effects are significant, and suggested that the heat transfer model assumed by Hord in the B-factor model variant is a poorer approximation. Watanabe et al. [19] simulated the heat transfer process between the bubble and the work mass using a one-dimensional unsteady-state thermal conductivity model. They analyzed the flow using the singularity method. Using an agent model, Goel et al. [20] synthesized the evaporation and condensation coefficients and the degree of influence of the thermophysical parameters on the objective function in the cavitation model. They gave optimal equation coefficients for the cavitation model. Tani et al. [21] applied the B-factor theory to correct the saturated vapor pressure variation brought about by temperature differences in the cavitation process and proposed a cavitation model considering thermodynamic effects. Tseng et al. [22] established an interface-based cavitation model, modified the model according to the low-temperature fluid medium, and investigated the thermodynamic effects by solving the energy equation in the full basin and combining the available physical parameters to verify the validity of the calculation method and the model. To study thermodynamic effects in different fluids at different temperatures, De et al. [23] proposed a stable semi-one-dimensional model based on the Rayleigh-Plesset equation to study the internal cavitation considering thermodynamic effects. Research on cavitation under thermodynamic effects has made some achievements, but it mainly focuses on inducer and airfoil flow cavitation. The research on the internal flow pattern and cavitation characteristics under a thermodynamic effect in small centrifugal pumps such as automobile electronic pumps remains to be further studied.

This study is focused on the cavitation flow field characteristics at different operating points of a high-speed automotive electronic water pump at a rated speed (5400 r/min), modifying the Singhal cavitation model considering the influence of thermodynamic effects, and carrying out numerical simulations of the cavitation full flow field at different temperatures (25 °C, 50 °C and 70 °C) based on the modified cavitation model to investigate the cavitation performance, bubble distribution law and pressure pulsation characteristics of an automotive electronic water pump at different temperatures, providing a theoretical basis for preventing and reducing the cavitation phenomenon of an automotive electronic pump.

## 2. Three-Dimensional Model and Grid Division

### 2.1. Model Pump Parameters

In this study, an automotive electronic water pump with a specific speed of 81 is used as the object. The working environment temperature of the pump is −40 °C to 80 °C, which is a micro high-speed pump driven by a DC brushless motor and controlled by an electronic unit. Table 1 shows the main parameters of the automotive electronic pump.

**Figure 1:**
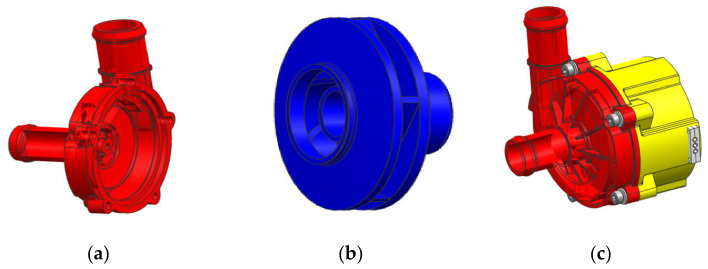
Structure diagram of automotive electronic water pump: (**a**) pump barrel, (**b**) impeller, and (**c**) motor base.

The automotive electronic water pump structure uses a closed centrifugal impeller and spiral pressurized volute as the main hydraulic components and an integral molding of the rotor and motor rotor. The whole pump body is divided into a pump barrel, impeller, and motor base, as shown in Figure 1.

### 2.2. Division of Computational Grid

**Figure 2:**
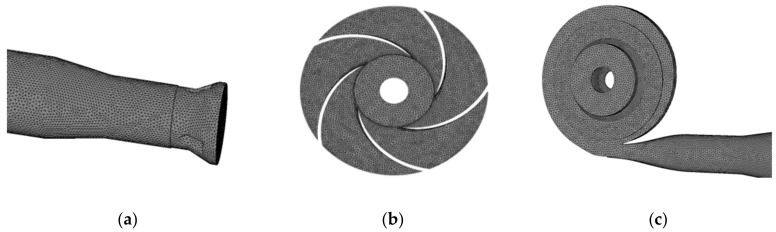
Grid of automotive electronic water pump: (**a**) inlet section, (**b**) impeller, and (**c**) volute and outlet.

**Figure 3:**
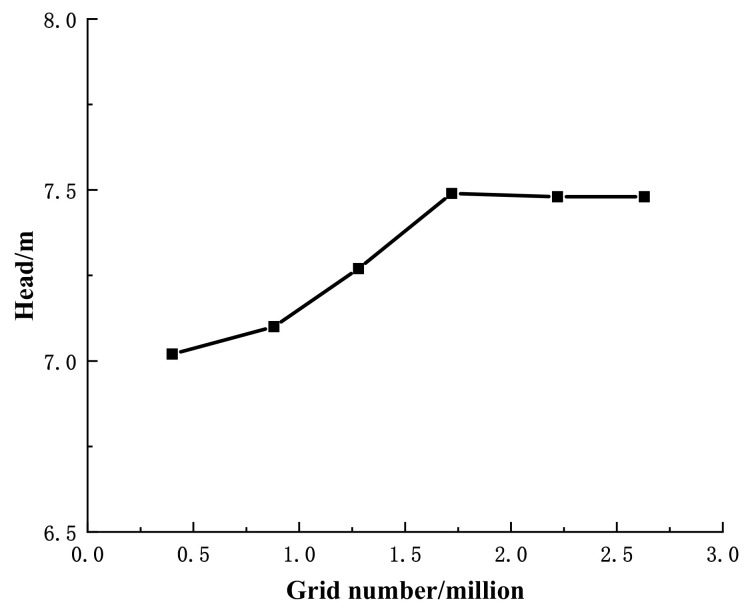
Grid independence analysis.

Due to the complex structure, ICEM CFD software was used for unstructured meshing of the computational domain of the main overcurrent components of the automotive electronic water pump, as shown in Figure 2. To consider the calculation cycle and its reliability of numerical calculation, grid-independent analysis was carried out, as shown in Figure 3. Since the previous literature [24,25] showed that if the absolute error of the predicted value of the two heads before and after is within 2%, then the influence of the mesh elements can be ignored. From Figure 3, when the grid number is less than 1.72 million, the grid number has a large influence on the head. When the grid number is larger than 1.72 million, the numerical calculation of the head varies by 1% with the increase of the grid number, and the final determination of the numerical calculation grid number is around 1.72 million. Grid information for parts of automotive electronic pumps is shown in Table 2.

## 3. Numerical Method

### 3.1. Turbulence Model

The corresponding Reynolds numbers at different temperatures in this study are 1.97 × 10^5^ (25 °C), 1.95 × 10^5^ (50 °C) and 1.93 × 1 0^5^ (70 °C), which are high Reynolds number cases. Based on the research experience and the literature analysis [26], the RNG *k-ε* turbulence model is chosen to calculate the cavitation. Its control equation is:

(1) $$\frac{\partial \left(\rho k\right)}{\partial t}\;+\;\nabla \;\cdot \;\left(\rho ku_{i}\right)\;=\;\nabla \;\cdot \;\left[\left(\mu \;+\;\frac{\mu_{t}}{\sigma_{k}}\right)\nabla k\right]\;+\;P_{t}-\rho \varepsilon$$

(2) $$\frac{\partial (\rho \varepsilon )}{\partial t}\;+\;\nabla \;\cdot \;\left(\rho \varepsilon u_{i}\right)\;=\;C_{\varepsilon 1}\frac{\varepsilon }{k}P_{t}\;-\;C_{\varepsilon 2}\rho \frac{\varepsilon^{2}}{k}\;+\;\nabla \;\cdot \;[(\mu \;+\;\frac{\mu_{t}}{\sigma_{\varepsilon }})\nabla \varepsilon ]$$

(3) $$\mu_{t}\;=\;\frac{C_{\mu }\rho k^{2}}{\varepsilon }$$

where *k* is turbulent kinetic energy; *ε* is turbulent dissipation term; *P*_t_ is turbulent kinetic energy generation term; $C_{\varepsilon 1}\;=\;1.42$, $C_{\varepsilon 2}\;=\;1.68$, $C_{\mu }\;=\;0.085$, $\sigma_{k}\;=\;0.7179$, $\sigma_{\varepsilon }\;=\;0.7179$.

### 3.2. Modification of Cavitation Model Considering Thermodynamic Effects

The commonly used cavitation simulation is based on the cavitation dynamics equation, namely the Rayleigh-Plesset equation [27]. The specific form is as follows:

(4) $$\frac{p_{B}\left(t\right)\;-\;p_{\infty }\left(t\right)}{\rho_{l}}\;=\;R_{B}\frac{d^{2}R_{B}}{dt^{2}}+\frac{3}{2}{\left(\frac{dR_{B}}{dt}\right)}^{2}\;+\;\frac{4\nu_{l}}{R}\frac{dR_{B}}{dt}\;+\;\frac{2S}{\rho_{l}R}$$

The left side of the equation is the pressure-driven term, determined by the vacuum’s boundary conditions. The right side of the equation shows the second-order motion term of the vacuole, the first-order motion term of the vacuole, the viscosity term, and the surface tension term, in that order, where *R*_B_ is the radius of the vacuole; $p_{B}\left(t\right)$ is the pressure inside the vacuole; $p_{\infty }\left(t\right)$ is the pressure at infinity; $\rho_{l}$ is the density of the liquid; is the kinematic viscosity of the liquid phase; S is the surface tension coefficient.

Based on this equation, the Singhal cavitation model [28] is derived by neglecting the second-order term, the viscous term, and the effect of surface tension of the equation:

When $p\;<\;p_{v}$, liquid vaporizes into bubbles

(5) $$m^{+}\;=\;C_{vap}\frac{3\alpha_{nuc}\left(1\;-\;\alpha_{\nu }\right)\rho_{\nu }}{R_{B}}\sqrt{\frac{2}{3}\frac{p_{B}\left(t\right)\;-\;p_{\infty }\left(t\right)}{\rho_{l}}}$$

When $p\;>\;p_{v}$, the cavity condenses into liquid

(6) $$m^{-}\;=\;C_{con}\frac{3\alpha_{\nu }\rho_{\nu }}{R_{B}}\sqrt{\frac{2}{3}\frac{p_{\infty }\left(t\right)\;-\;p_{B}\left(t\right)}{\rho_{l}}}$$

where $C_{vap}$ and $C_{con}$ are the correction factors for the vaporization and condensation source terms, respectively, $\alpha_{nuc}$ is the volume fraction of the cavitation nucleus, and their values are $C_{vap}\;=\;50$, $C_{con}\;=\;0.01$, $\alpha_{nuc}\;=\;5\;\times \;10^{4}$.

Singhal cavitation model is derived based on the isothermal assumption, ignoring the effect of thermodynamic effects in cavitation. When cavitation occurs, the liquid vaporization absorbs the latent heat of vaporization, resulting in a decrease in temperature near the vacuole, and a certain temperature difference is formed inside and outside the vacuole. The temperature difference affects the growth of the vacuole. Moreover, the Singhal cavitation model is modified in this paper to consider the thermodynamic effect of cavitation under different temperature conditions. The second-order term, viscous term, and surface tension of the Rayleigh-Plesset equation is neglected, and the Taylor series expansion of cavitation pressure is carried out to retain the first-order term. The second-order term and other terms after they are neglected [29], we can obtain the following:

(7) $$\frac{dR_{B}}{dt}\;=\;\sqrt{\frac{2}{3}\left[\frac{p_{B}\left(t\right)\;-\;p_{\infty }\left(t\right)}{\rho_{l}}\;+\;\frac{dp_{B}}{dT}\frac{T_{B}\;-\;T_{\infty }}{\rho_{l}}\right]}$$

From the Clapeyron-Clausius equation, we can obtain:

(8) $$\frac{dp_{B}}{dT}\;=\;\frac{\rho_{l}\rho_{B}}{T\left(\rho_{l}\;-\;\rho_{B}\right)}L$$

where *L* is the latent heat of vaporization.

Substituting Equation (8) into Equation (7) yields:

(9) $$\frac{dR_{B}}{dt}\;=\;\sqrt{\frac{2}{3}\left[\frac{p_{B}\left(t\right)\;-\;p_{\infty }\left(t\right)}{\rho_{l}}\;+\;\left(\frac{\rho_{l}\rho_{B}}{T\left(\rho_{l}-\rho_{B}\right)}L\right)\frac{T_{B}\;-\;T_{\infty }}{\rho_{l}}\right]}$$

From Equations (5), (6) and (9), the condensation source term and the vaporization source term of the cavitation model considering thermodynamic effects are:

(10) $$m^{+}\;=\;C_{vap}\frac{3\alpha_{nuc}\left(1\;-\;\alpha_{\nu }\right)\rho_{\nu }}{R_{B}}\sqrt{\frac{2}{3}\left[\frac{p_{B}\left(t\right)\;-\;p_{\infty }\left(t\right)}{\rho_{l}}\;+\;\left(\frac{\rho_{l}\rho_{B}}{T\left(\rho_{l}\;-\;\rho_{B}\right)}L\right)\frac{T_{B}-T_{\infty }}{\rho_{l}}\right]}$$

(11) $$m^{-}\;=\;C_{con}\frac{3\alpha_{\nu }\rho_{\nu }}{R_{B}}\sqrt{\frac{2}{3}\left[\frac{p_{\infty }\left(t\right)\;-\;p_{B}\left(t\right)}{\rho_{l}}\;+\;\left(\frac{\rho_{l}\rho_{B}}{T\left(\rho_{l}\;-\;\rho_{B}\right)}L\right)\frac{T_{B}\;-\;T_{\infty }}{\rho_{l}}\right]}$$

### 3.3. Boundary Condition Setting

CFX software was used to simulate the flow field of the automotive electronic water pump at different temperatures with pure water as the medium for single-phase steady simulation and two-phase flow simulation with the addition of cavitation model. The RNG k-ε turbulence model was used for each phase of the simulation, the simulation speed was 5400 r/min, the inlet and outlet boundary conditions were set for the total pressure inlet and mass flow outlet, the cross-interface connection of GGI was used, and the convergence accuracy of all simulations was set to 1 × 10^−4^.

For the single-phase stability simulation, the dynamic-static interface is set as the frozen rotor and the number of iterations is 1000. For steady cavitation simulation, the modified cavitation model is written into the CFX using the CEL language, pure water at the corresponding temperature is added as the working medium, and water vapor is added as the bubble generated during cavitation. The calculation sets the volume fraction of the liquid phase at the inlet boundary to 1 and the volume fraction of the vapor phase to 0. The inlet pressure is adjusted downwards by 0.2 atm for each simulation, and the change in the head is compared to determine the inlet pressure at which cavitation occurs. The physical parameters of pure water and water vapor at different temperatures are shown in Table 3. It can be seen that the temperature has a large effect on the physical parameters of water density, dynamic viscosity, and vaporization pressure, and the value of each physical parameter is smaller for water vapor compared with water.

To obtain a stable and reliable solution, the unsteady simulation of the cavitation flow field takes its steady simulation results at the corresponding temperature as the initial conditions. The dynamic-static intersection is modified to a transient rotor stator in the calculation; the impeller rotation of 360° is taken as a calculation cycle, the time step is set to 6.17 × 10^−5^ s (calculated for every 2° rotation of the impeller), the number of inner loops at each time step is 20, and the total calculation time is set to 0.2 s (18 rotation periods). The Courant number is used as a criterion to determine whether the time step satisfies the periodic numerical simulation, and is defined as:

(12) $$C_{0}\;=\;v\frac{\Delta t}{L}\;<\;100$$

where L is the smallest size of the grid cell, v is the biggest velocity of the main flow, and Δt is the time step. The maximum $C_{0}$ obtained by the calculation is 5.23, which satisfies the independence of the time step.

### 3.4. Monitoring Point Setting

**Figure 4:**
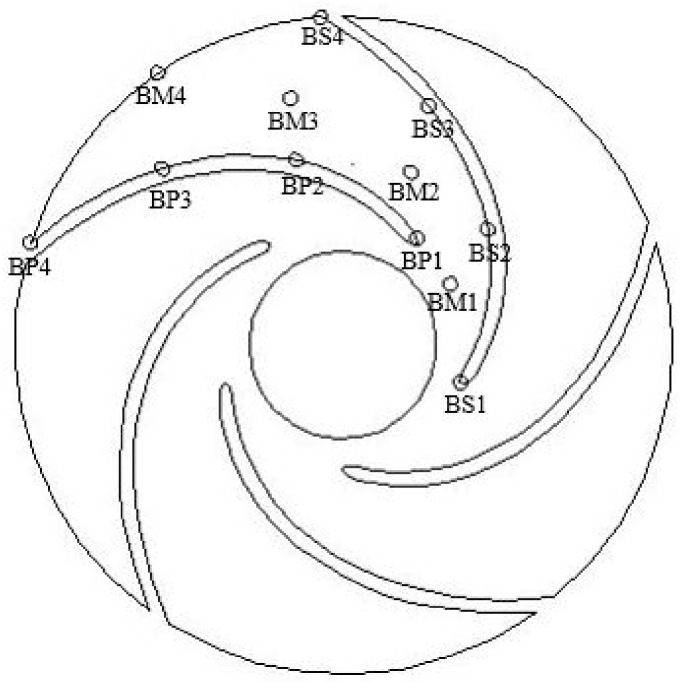
Monitoring points position in an automotive electronic water pump.

To study the pressure variation inside the impeller of an automotive electronic water pump during cavitation under thermal effects, 12 monitoring points are set up at the impeller position of the pump, as shown in Figure 4.

## 4. Analysis of Internal Flow of Automotive Electronic Water Pump under Non-Cavitation Condition

### 4.1. Verification of External Characteristics by Numerical Calculation

**Figure 5:**
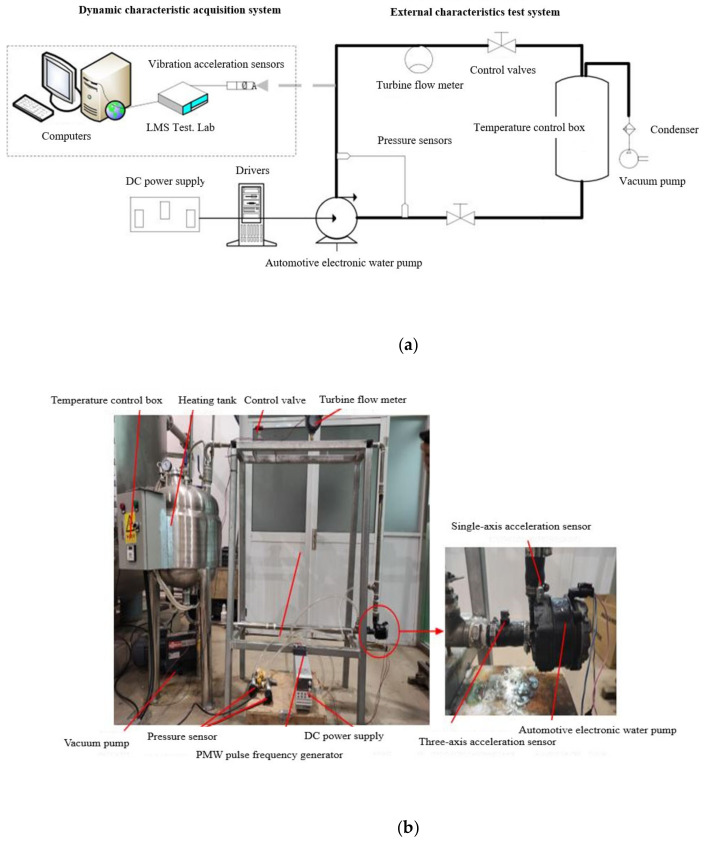
Test bench for an automotive electronic water pump: (**a**) experimental schematic, (**b**) experimental loop.

The external characteristic curve is the external expression of the internal flow characteristics of the automotive electronic water pump. Numerical calculations are performed for the automotive electronic water pump at different flow rates, and tests verify the reliability of the numerical calculation results. The performance test of an automotive electronic water pump is carried out concerning GB/T 3216–2016, and the test bench is shown in Figure 5.

Using the head coefficient *ψ* to calculate the external characteristics, the head coefficient can be calculated as the following equation:

(13) $$\psi \;=\;\frac{gH}{u_{2}^{2}/2}$$

where $u_{2}$ is the circumferential exit speed of the impeller.

**Figure 6:**
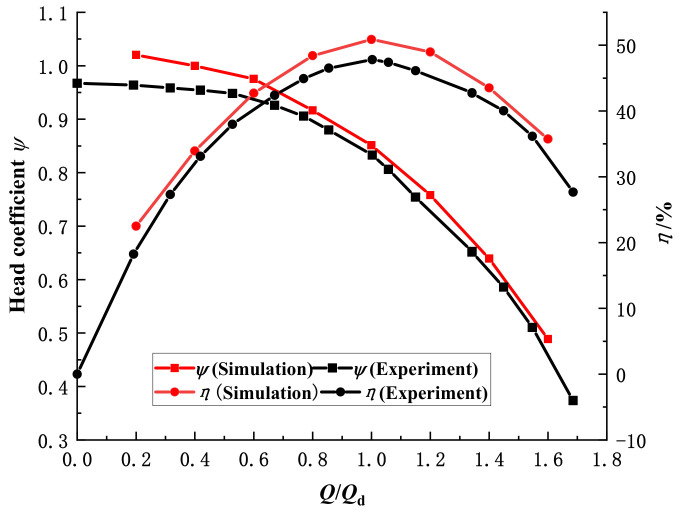
Comparison of pump performance between numerical and experimental results.

Figure 6 shows a comparison of the external characteristics results. As can be seen from the figure, the experimental head coefficient and efficiency are slightly lower than the numerical calculation due to the simplification of the model during the numerical calculation for the same flow conditions. At a small flow rate (0.2*Q*_d_), the difference between the simulated and experimental values of the head coefficient is 0.06; at 0.8*Q*_d_~1.6*Q*_d_, the two curves are in good agreement, and it can be considered that the numerical calculation method can accurately predict the external characteristics of the pump and ensure the accuracy of the subsequent analysis.

### 4.2. Pressure Field Distribution in the Pump at Different Temperatures

**Figure 7:**
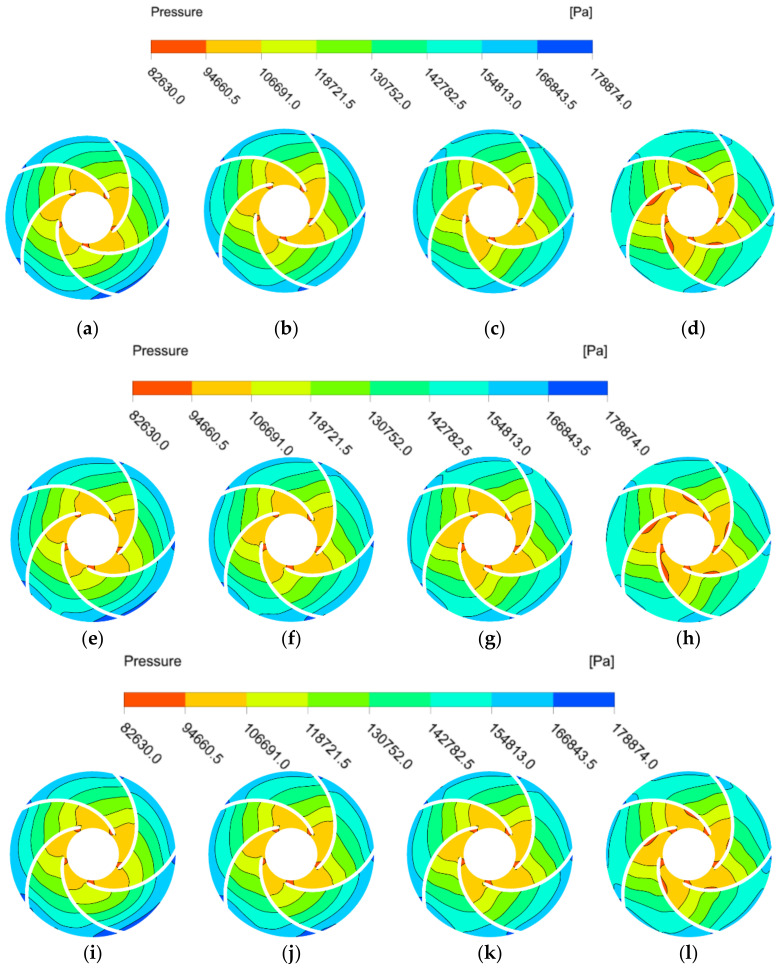
Pressure distribution of impeller under at different temperatures. (**a**) 0.6Q_d_ at 25 °C; (**b**) 0.8*Q*_d_ at 25 °C; (**c**) 1.0*Q*_d_ at 25 °C; (**d**) 1.2*Q*_d_ at 25 °C; (**e**) 0.6*Q*_d_ at 50 °C; (**f**) 0.8*Q*_d_ at 50 °C; (**g**) 1.0*Q*_d_ at 50 °C; (**h**) 1.2*Q*_d_ at 50 °C; (**i**) 0.6*Q*_d_ at 70 °C; (**j**) 0.8*Q*_d_ at 70 °C; (**k**) 1.0*Q*_d_ at 70 °C; (**l**) 1.2*Q*_d_ at 70 °C.

Figure 7 shows the static pressure distribution clouds of the impeller of the automotive electronic water pump under four different flow conditions at 25 °C, 50 °C, and 70 °C (speed of 5400 r/min). The static pressure distribution at different temperatures is similar; from the blade inlet to the blade outlet, the pressure in the impeller is of a gradient-type growth, and the pressure distribution is more uniform. As the flow rate increases, the blade suction surface pressure gradually decreases, and the low-pressure area gradually increases. Under the large flow condition (1.2*Q*_d_), the area of the low-pressure area at the inlet gradually decreases as the temperature increases.

## 5. Automotive Electronic Water Pump Cavitation Performance Analysis

### 5.1. Computational Analysis of Cavitation Performance Considering Thermodynamic Effects

The net positive suction head is the surplus energy per unit weight of water at the pump suction inlet that exceeds the vaporization pressure and is expressed as follows:

(14) $$\mathrm{NPSHa}\;=\;\frac{p_{1}\;-\;p_{v}}{\rho g}$$

where $p_{1}$ is the automotive electronic water pump inlet pressure; $p_{v}$ is the saturation vapor pressure of water at that temperature; ρ is the fluid density.

**Figure 8:**
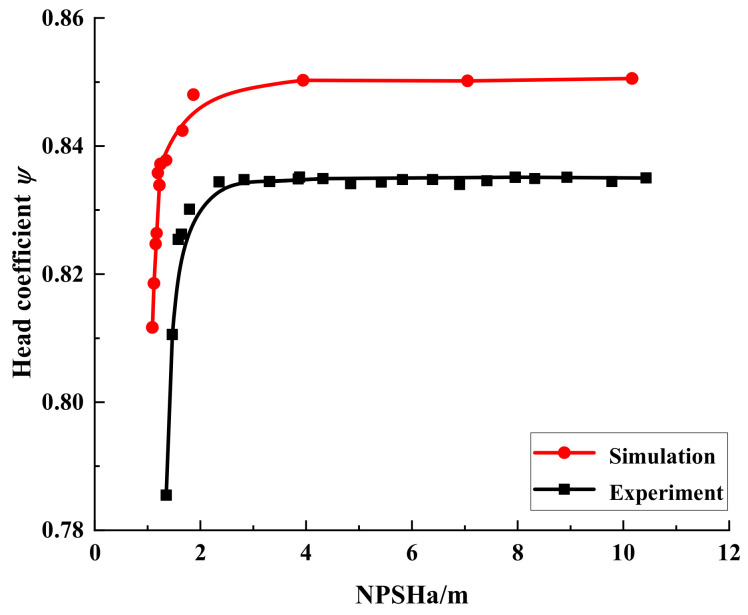
Comparison of cavitation performance curves between numerical and experimental results.

Figure 8 shows the cavitation performance curves of the simulated and tested automotive electronic water pump at a temperature of 25 °C (flow rate of 1.25 m^3^/h and speed of 5400 r/min). As can be seen from the figure, the head coefficient of the test value is smaller than the simulated value under the same inlet conditions, which are related to the existence of friction between the fluid and the pipe wall and energy loss between the motor and the impeller. Overall, the simulated and experimental cavitation performance curves have the same stagnation point position and trend. It can be considered that the numerical calculation method can accurately predict the cavitation performance of the pump and ensure the accuracy of subsequent cavitation calculations.

**Figure 9:**
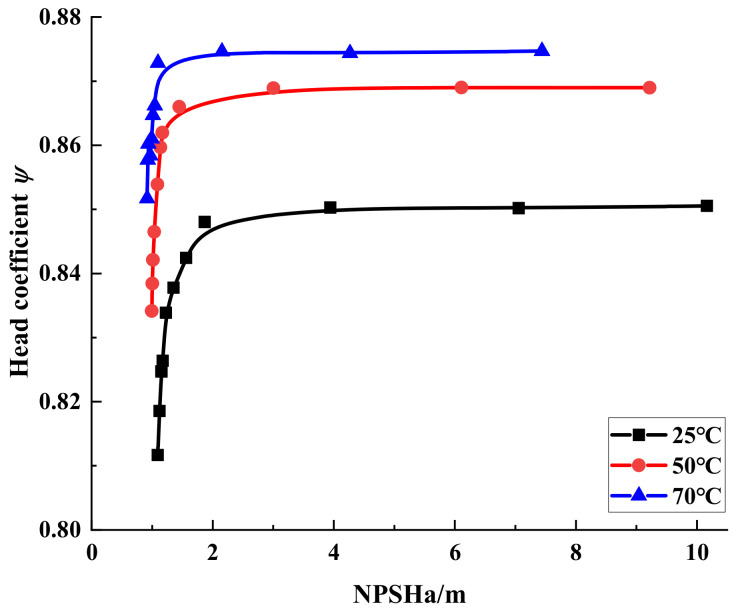
Cavitation performance curves with different temperatures.

Figure 9 shows the cavitation performance curves of the simulated automotive electronic water pump at three different temperatures (flow rate of 1.25 m^3^/h and speed of 5400 r/min). We define the automotive electronic water pump head drop of 1% as incipient cavitation, a head drop of 3% as cavitation, and the NPSH corresponding to the head drop of 3% as the required net positive suction head of the pump (NPSHr). The NPSHrs at three temperatures were 1.15 m at 25 °C, 1.01 m at 50 °C, and 0.91 m at 70°C. As can be seen from the figure, with the decrease of NPSHa, the head coefficient maintains a level for a period of time and then rapidly decreases, which is because the flow pattern inside the automotive electronic water pump is turbulent and sensitive to cavitation, and the hydraulic performance deteriorates sharply once cavitation occurs; with the increase of temperature, NPSHr gradually decreases, and the anti-cavitation performance of the automotive electronic water pump gradually increases.

### 5.2. Cavitation Steady Analysis Considering Thermodynamic Effects

#### 5.2.1. Bubble Shape during Cavitation Formation

**Figure 10:**
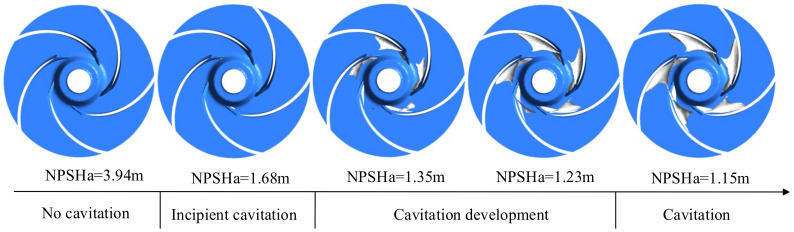
Bubble distribution during the cavitation formation process.

The bubble is the most visual product when cavitation occurs, and the volume fraction of the bubble can judge the degree of cavitation. Figure 10 shows the distribution of bubbles in the impeller at different NPSHa for a bubble volume concentration of 10% at a temperature of 25 °C (flow rate of 1.25 m^3^/h and speed of 5400 r/min) for an automotive electronic water pump. It can be seen from the figure that when the NPSHa is larger, no bubble is generated in the impeller; when the NPSHa is reduced to 1.68 m, a small number of bubbles appears at the inlet side of the impeller near the suction surface of the blade, which is the cavitation inception stage. As the NPSHa gradually decreases, the distribution area of the bubble starts to spread from the suction surface to the pressure surface, and cavitation further develops; when the NPSHa decreases to NPSHr, the bubbles gradually increase and develop to the pressure surface and start to block the impeller flow channel, and the high-speed jet caused by the rupture of the bubbles will damage the impeller and produce cavitation, resulting in a serious drop in the head.

#### 5.2.2. Analysis of the Pressure Field during Cavitation Formation

**Figure 11:**
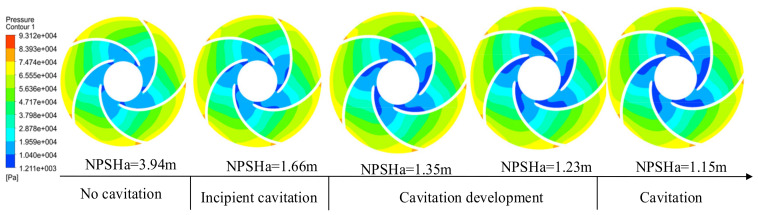
Pressure distribution of bubble formation process.

The occurrence of cavitation is pressure-dependent, and its distribution is influenced by the law of hydrostatic pressure distribution. Figure 11 shows cloud plots of static pressure distributions at different NPSHas of an automotive electronic water pump at a temperature of 25 °C (flow rate 1.25 m^3^/h, speed 5400 r/min). As can be seen from the figure, from the blade inlet to the blade outlet, the pressure gradually increased; five blades on the static pressure distribution show a similar pattern in which the static pressure of the fluid along with the fluid flow direction increased. In the blade outlet pressure, it reached the maximum. The range of local low-pressure areas gradually expands with the decrease of NPSHa. In the no cavitation and incipient cavitation stage, the influence of NPSHa on the size of the low-pressure area range is small; from the cavitation development stage to the cavitation stage, the influence of NPSHa size on the size of the low-pressure area range at the impeller inlet of the automotive electronic water pump is large. Because of the low pressure at the impeller inlet, cavitation generally occurs first from the suction surface of the impeller inlet blade.

#### 5.2.3. Cavitation Flow Field Analysis

**Figure 12:**
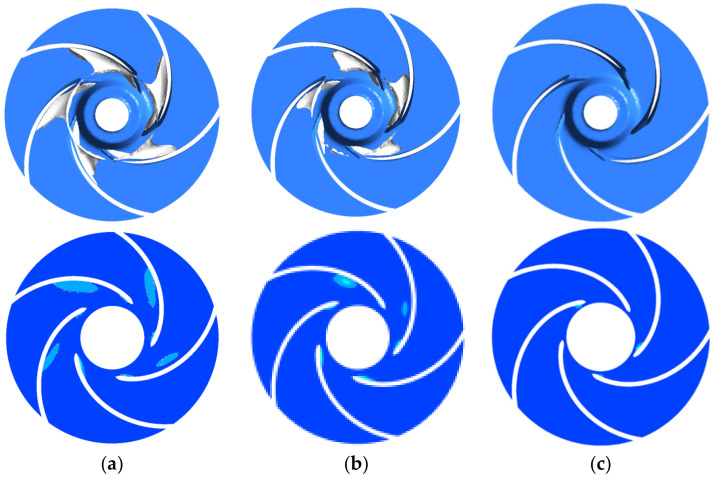
Bubble distribution with different temperatures based on the same. (**a**) T = 25 ℃; (**b**) T = 50 ℃; (**c**) T = 70 ℃.

Figure 12 shows the bubble distribution inside the impeller at three temperatures when the NPSHa is 1.15 m (flow rate 1.25 m^3^/h, speed 5400 r/min). At 70 °C, when the inlet pressure is 0.407 atm, some bubbles are produced near the suction surface of some of the impeller’s blades, and the pump head does not drop significantly at this time. At 50 °C, when the inlet pressure is 0.221 atm, the bubble area becomes larger, and all impeller blades produce bubbles and development to the middle of the radial direction of the impeller; at this time, the pump head drops by 1.5%. It can be seen that the bubbles generated at this time are enough to impact the performance of the pump. At 25 °C, when the inlet pressure is 0.131 atm, the bubbles cluster continues to increase and begins to block the flow channel, at which time the pump head decreases by 3%, and cavitation occurs. At 25 °C, the low-pressure area at the impeller inlet suction surface is larger, and as the temperature rises, the area of the low-pressure area becomes smaller and smaller, and the area of the corresponding bubbles also gradually decreases. In addition, both the low-pressure area and the bubble cluster are axially distributed.

Combining Figure 8 and Figure 9, it is found that the number of bubbles is higher during the stage of a sharp decline in the head, and the bubbles have a certain influence on the head; the bubbles are all generated from near the low-pressure area at the blade inlet and develop along with the impeller outlet, and the temperature has a certain influence on the distribution of bubble in the automotive electronic water pump. The bubble distribution inside the impeller is consistent with the cavitation performance curve: the more bubbles are generated, the more obvious the drop of its head.

### 5.3. Cavitation Transient Analysis Considering Thermodynamic Effects

#### 5.3.1. Transient Bubble in the Impeller

**Figure 13:**
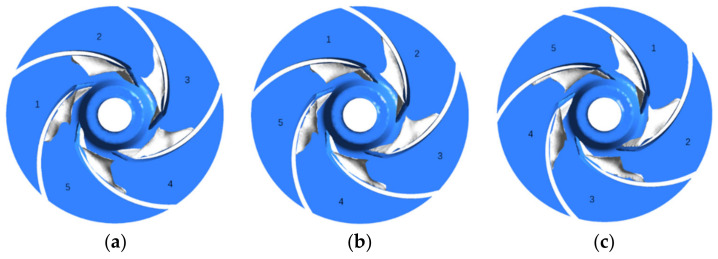

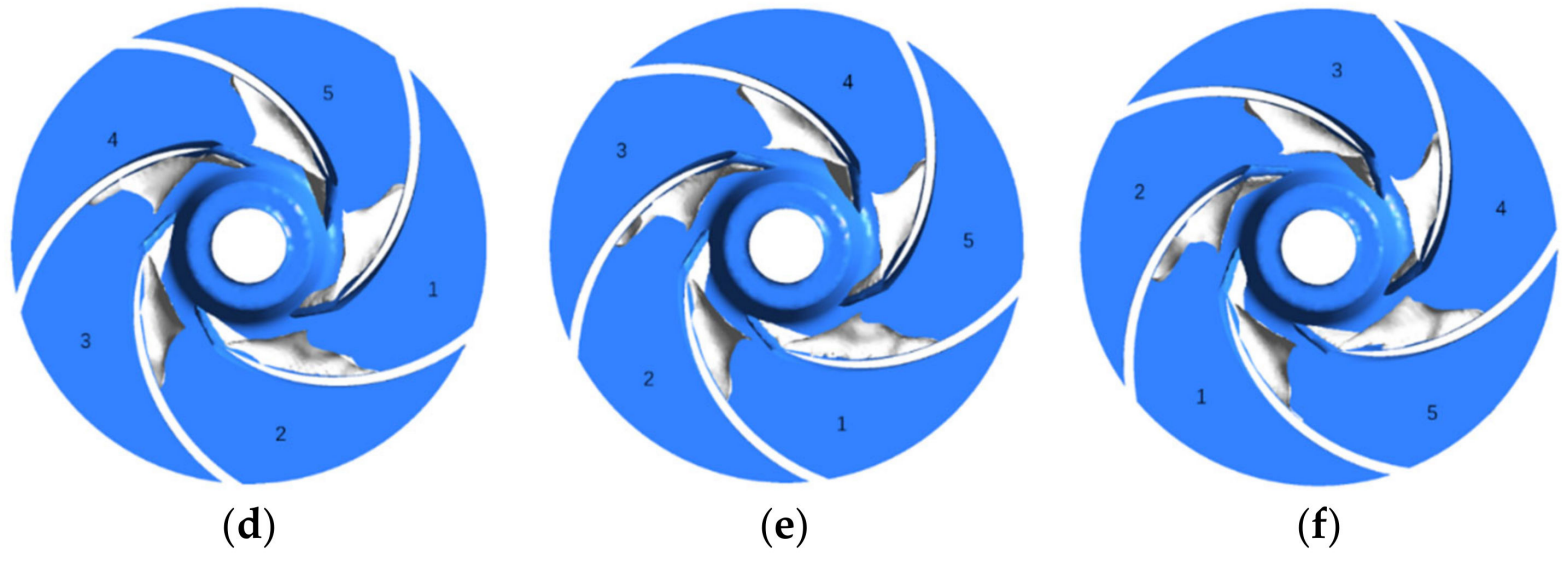
Bubble distribution in the impeller domain. (**a**) *t* = 0; (**b**) *t* = 1/6 T; (**c**) *t* = 2/6 T; (**d**) *t* = 3/6 T; (**e**) *t* = 4/6 T; (**f**) *t* = 5/6 T.

Figure 13 shows the distribution of bubbles at a temperature of 25 °C and an inlet pressure of 0.131 atm for an impeller with a vapor volume concentration of 10% at six different moments in a cycle. It can be seen from the figure that at the moment t = 0, the suction surface in each flow channel adheres to a continuous bubble, and the shape of the bubble is of an irregular rugby ball with a wide middle and narrow front and back; as the blades are periodically arranged, with the impeller rotation, the bubble change in each flow channel has a similar pattern. Take runner No. 1 as an example: at *t* = 2/6 T, the bubble in the middle of the flow channel will be detached; at *t* = 3/6 T, the volume of the bubble in runner No. 1 is smaller than at *t* = 2/6 T. At this time, the bubble has detached and collapsed in the high-pressure area; with the development of time, at *t* = 3/6 T to *t* = 5/6 T, the bubble in runner No. 1 slowly develops toward the end and detaches. On the whole, the detachment and collapse of the bubble occurred in the middle and tail of the bubble cluster, where the middle bubble detached less and the tail detached more; the changes in the position and shape of the bubble were relatively small, indicating that the whole process of cavitation was relatively stable at this time.

#### 5.3.2. Comparative Analysis of Pressure Pulsation in the Frequency Domain

To analyze the relative pressure variation at each monitoring point within the impeller, we use a pressure coefficient expressed as:

(15) $$C_{p}^{*}\;=\;\frac{p\;-\;\bar{p}}{0.5\rho u_{2}^{2}}$$

where p is the instantaneous pressure, $\bar{p}$ is the average pressure, ρ is the fluid density, and $u_{2}$ is the circumferential velocity at the impeller outlet. $\bar{p}$ is the average pressure of the last eight cycles simulated at the monitoring points in this study.

**Figure 14:**
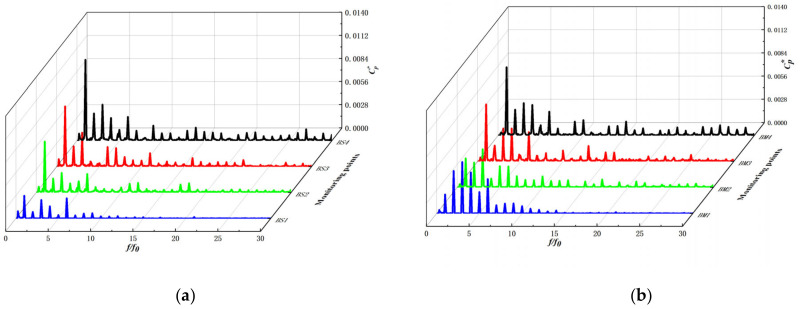

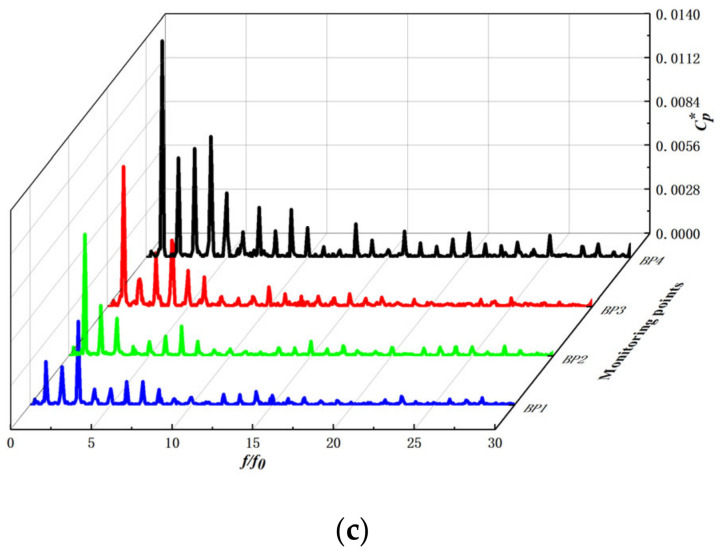
Frequency characteristic in the impeller. (**a**) Blade suction surface; (**b**) Middle of the flow channel; (**c**) Blade pressure surface.

Figure 14 shows the frequency domain of the pressure pulsation at the monitoring point inside the impeller at 25 °C (flow rate 1.25 m^3^/h, speed 5400 r/min) during a no cavitation operating condition of the automotive electronic water pump. As can be seen from the figure, along with the impeller runner from the blade inlet to the blade outlet, the pressure pulsation amplitude at each monitoring point gradually increases, the amplitude frequency is the axial frequency and its multiplier frequency, and the pressure pulsation amplitude reaches the maximum at the axial frequency. At the axial frequency, the pressure pulsation amplitude from the blade suction surface to the blade pressure surface pressure pulsation amplitude gradually increases; at the impeller runner inlet, the blade suction surface (BS1) and pressure surface (BP1) pressure pulsation amplitude is higher than the middle of the impeller runner (BM1), which is because at the impeller inlet, the three-jaw ring disturbs the incoming flow field and intensifies the pressure fluctuation at the impeller inlet.

**Figure 15:**
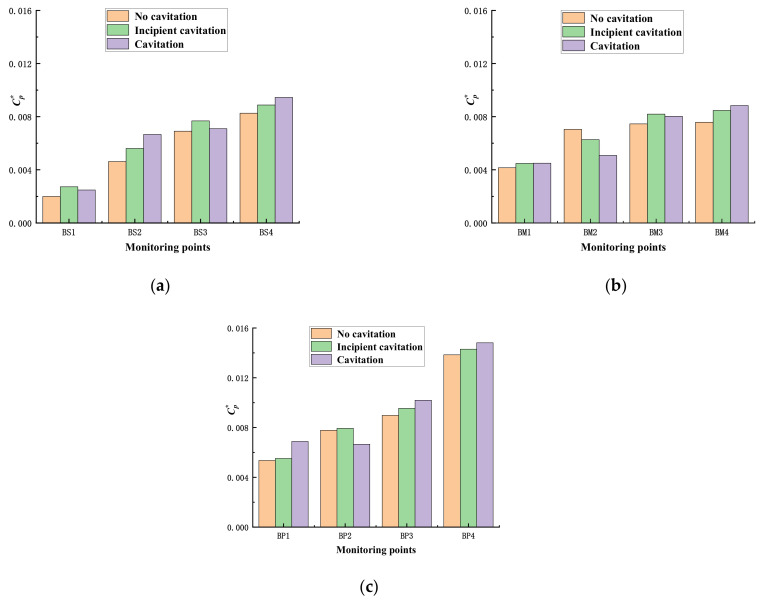
The maximum amplitude of pressure fluctuations in the impeller. (**a**) Blade suction surface; (**b**) Middle of the flow channel; (**c**) Blade pressure surface.

Figure 15 shows the maximum values of pressure pulsations at the monitoring points inside the impeller during no cavitation, incipient cavitation, and cavitation of the automotive electronic water pump. The maximum amplitude value increases gradually along the fluid flow direction, except for individual monitoring points, and reaches the maximum value at the exit of each working surface. Overall, the maximum value of pressure pulsation during cavitation is higher on the blade working surface than no cavitation, which may be related to the generation and collapse of bubbles.

## 6. Conclusions

In this study, we have considered the influence of thermodynamic effect on the cavitation performance of hydromechanics, corrected the Singhal cavitation model considering a thermodynamic effect, written the corrected model into CFX software for numerical simulation of cavitation of automotive electronic water pump, explored the cavitation performance and the distribution pattern of cavitation bubble of automotive electronic water pump under different temperature (25 °C, 50 °C, 70 °C), and drawn the following conclusions:

1. The static pressure distribution of the automotive electronic water pump is similar at different temperatures. When operating under different flow conditions, there is a local low-pressure area on the suction surface of the blade that is more prone to cavitation, and the range of the low-pressure area gradually increases as the flow rate increases. Under the large flow condition (1.2*Q*_d_), the area of the low-pressure area at the inlet gradually decreases as the temperature increases.
2. An automotive electronic water pump is sensitive to cavitation; once cavitation occurs will lead to a sharp deterioration in hydraulic performance. The required net positive suction head will decrease with the increase in temperature; the higher the temperature of the automotive electronic water pump’s anti-cavitation performance, the less likely cavitation is.
3. When the inlet pressure of the automotive electronic water pump is large, no cavitation is generated inside the impeller. When the inlet pressure decreases to a certain value, the bubble starts to be generated on the suction surface of the impeller. It gradually increases with the decrease of the inlet pressure. The impeller’s five blades in the low-pressure area and the bubble cluster show the same pattern, and the expansion of the bubble cluster and the expansion in the low-pressure area of the impeller show the same trend. The pressure is closely related to the generation and development of cavitation.
4. Along with the impeller runner from the blade inlet to the blade outlet, each monitoring point’s pressure pulsation amplitude gradually increased, and the amplitude frequency of the axial frequency and its multiplier frequency, as well as the pressure pulsation amplitude at the axial frequency, reached the maximum. In the high-frequency region, the pressure pulsation amplitude at the blade suction surface, the middle of the flow channel, and the blade pressure surface gradually increases along the inlet to the outlet. From no cavitation to cavitation, the maximum value of the pressure pulsation at the working surface gradually increases and reaches a maximum at the outlet.
5. This study is focused on the cavitation flow field characteristics at different operating points of a high-speed automotive electronic water pump at a rated speed (5400 r/min). In order to better prevent and reduce the cavitation phenomenon of the automotive electronic pump, our future research will focus on the cavitation monitoring technology, and we will establish a cavitation intelligent monitoring system to monitor the operating status of the automotive electronic water pump in real time.

## Figures and Tables

**Table 1:** Automotive electronic water pump parameters.

Parameters	Value
Rated flow rate, *Q*_d_	1.25 m^3^/h
Rated head, *H*_d_	7 54 m
Rated rotating speed, *n*	5400 r/min
Specific speed, *n_s_*	81
Blade number, *Z*	5
Inlet diameter of impeller, *D*_j_	17 mm
Outlet diameter of impeller, *D*_2_	47 mm

**Table 2:** Grid information of domains.

Domains	Inlet Section	Impeller	Volute and Outlet	Total
Grid number	328,403	562,914	833,778	1,725,095
Node number	57,584	96,006	143,978	297,568
Grid quality	0.34	0.32	0.26	/
y +	19.4145	38.9787	54.9447	/

**Table 3:** Physical parameters of water and water vapor at different temperatures.

Physical Parameters	25 °C		50 °C		70 °C	
	Water	Water Vapor	Water	Water Vapor	Water	Water Vapor
Density (kg/m^3^)	997	0.02308	988.1	0.08302	977.8	0.1982
dynamic viscosity (10^5^ Pa s)	8899	0.98626	54.94	1.002	40.16	1.0817
Constant pressure specific heat capacity (kJ/(kg K))	4.1817	1.9116	4.174	1.9343	4178	1962.7
Thermal conductivity(w/(m K))	0.6069	0.01854	0.6478	0.02182	0.6676	0.02346
Vaporization pressure (kPa)	3.1684	/	12.335	/	31.160	/

## Data Availability

The data supporting this study’s findings are available within the article. The data pressented in this study are available on request from the corresponding author.
